# Tetanus1Read before the meeting of the Pennsylvania State Veterinary Medical Association, Philadelphia, March 2, 1897.

**Published:** 1897-05

**Authors:** Leonard Pearson

**Affiliations:** Philadelphia, Pa.


### TETANUS.[1]

By Leonard Pearson, V.M.D.,
PHILADELPHIA, PA.

Tetanus of horses is a disease that has been known since the earliest times, and was described by Apsyrtus during the fourth century. The views in regard to this disease that have prevailed at various times have differed widely, and it has variously been ascribed to an "inflammation of the blood," an injury to the brain or spinal cord, a peculiar electrical condition of the atmosphere, foods of various kinds, etc. Subsequently tetanus was divided by the writers of text-books into several varieties, particularly traumatic, rheumatic, idiopathic; and each of these forms of disease was ascribed to different causes. It was not, however, until the researches of Nicolaier that the real cause of tetanus was discovered. Nicolaier's first announcements were published in 1884, and they were amply supported and confirmed by the researches of Kitasato, published in 1889. Kitasato was successful in isolating and in growing, in pure cultures, the tetanus bacillus, and he proved by the inoculation of animals that the disease could actually be produced by the bacillus that he had isolated and described.

The tetanus bacillus is an organism of rather peculiar shape. It is rather long and slender, and has a round enlargement at one end, which contains a spore. This bacillus has been found in earth, in pus, in manure, and in the secretion from infected wounds. The bacillus is isolated in the laboratory with much difficulty, because it will only grow where there is an absence of oxygen.

The spore of the tetanus bacillus is quite resistant, very much more so than the bacillus itself, and it requires heating at boiling temperature from two to five minutes to destroy it. The isolation of the germ in the laboratory is based upon this characteristic, and is carried out after the method first described and employed by Kitasato. A small quantity of the material to be examined is exposed for an hour at a temperature of 80° C., and in this way all of the bacteria and most of the spores in the substance, with the exception of the spores of tetanus, are destroyed, and the inoculation of culture media with material so treated enables one to obtain a pure culture of the tetanus bacillus without much further difficulty. The tetanus bacillus, and particularly its

[1] Read before the meeting of the Pennsylvania State Veterinary Medical Association, Philadelphia, March 2, 1897.

spores, is not only resistant to heat, but to other conditions that are unfavorable to the growth of germs. For instance, it requires exposure to a 5 per cent. creolin solution for five minutes or to 5 per cent. carbolic acid solution for ten hours to destroy it. A 5 per cent. carbolic acid solution, together with 0.5 per cent. of hydrochloric acid, destroys these organisms in twenty-five minutes. A particularly strong antiseptic for the spores of the tetanus bacillus is trichloride of iodine. This fact was discovered by Behring, and has been made use of in a number of important experiments. These germs are not only resistant to the digestive fluids, but they may even multiply in the digestive tract. This accounts for their presence in the manure of horses.

The tetanus bacilli are so widely distributed, and find the conditions outside of the living body so favorable to their existence, that opportunity for infection is abundant, and there are few places that are entirely free from this disease, although it is far more prevalent in some localities than in others. The manner in which the bacillus is introduced into the living animal is a matter of considerable moment as regards the danger of such inoculation, since the organism will not grow in the presence of oxygen ; all inoculations with the tetanus bacillus are not dangerous, but when the organism is introduced deeply into the tissues away from the air, or when it is introduced with other germs that use up the oxygen in the surrounding parts, tetanus may develop.

The wounds that are especially apt to be followed by the development of tetanus are wounds made by objects that have been in contact with the earth, and are therefore most apt to contain tetanus germs. For instance, pricking of the foot is especially dangerous in this respect, and a calk-wound of the coronet is also accompanied by considerable danger, as it is likely to be made by an object that has been in contact with earth and manure, and to be coated with more or less tetanus bacilli. A deep puncture with a piece of wood, castration, and docking have also been followed in many cases by tetanus. The use of clamps in castration was frequently followed by tetanus in the olden times, when it was not customary to disinfect these instruments. The infectious wound-secretion containing the bacilli and spores of tetanus would in this way be carried from one colt to another, and the death of a large proportion of the animals castrated by certain operators or in certain districts was not uncommon. The not rare cases of tetanus that occur without an external injury are explained by the assumption that infection has taken place through a wound that was so small as to escape notice, or that infection occurred through the mucous membrane of the intestinal canal.

The disease, tetanus, is caused by the tetanus bacillus by means of a toxin, or ptomaïn, that is developed by this germ. This substance is produced by the germs at the point of inoculation. It is carried by the circulation to all parts of the body, and its action on the central nervous system is such as to produce the muscular spasms that are characteristic of the disease. The toxin of tetanus is also produced when the germ is grown in culture-media in the laboratory, and it has been found that the injection of a small quantity of toxin that is entirely free from living organisms will produce the symptoms of tetanus. The power of this toxin is extraordinary, and but a very small quantity of it will produce death.

The rapidity also with which the toxin is developed is astonishing. Kitasato has found, for instance, that when mice are inoculated at the root of the tail, and afterward the skin and subcutaneous tissues around the inoculation are removed and cauterized, the treatment is without avail unless it is performed within one hour after the inoculation. In cases of tetanus toxin is found in the muscle-juice, the blood, the urine, and in the milk. As the toxin gradually passes from the point of inoculation, where it is developed, into the general circulation, it is noticed that rigidity of the muscles begins in the inoculated member or part of the body and gradually extends to the more distant portions. It has been shown by Nocard that by the time the symptoms of tetanus appear in the horse the saturation of the tissues with tetanus toxins is quite general, and the amount of toxin contained in the body at that time is enormous.

The bacilli themselves never enter the blood, and, of course, cannot pass into the circulation, and after they have elaborated enough toxin to cause death the post-mortem appearances, as is well known, are about normal, with the exception of the suppurating wound at the point of inoculation and more or less congestion of the central nervous system.

It has been shown by experiments conducted by Behring and Kitasato that the trichloride of iodine restricts to a very great extent the vitality and virulence of the tetanus bacillus, and by introducing into susceptible animals small quantities of tetanus cultures that have been treated with this agent, and gradually increasing the strength of such cultures by using smaller quantities of the trichloride of iodine, a certain degree of immunity can in time be developed, so that at last very large quantities of pure culture can be introduced without producing tetanus. An animal treated in this way gradually develops in its blood a chemical substance that neutralizes the effect of the toxin of tetanus, and which is, therefore, known as an antitoxin.

The immunity that the animal possesses depends upon the antitoxin that is present in its blood. If another susceptible animal is inoculated with a fatal dose of tetanus toxin, and is treated immediately thereafter with an adequate amount of antitoxin derived from the serum of the immunized animal, the development of the disease will in this way be prevented, and it is upon this principle that the treatment of tetanus with antitoxin is based. Experiments conducted by Vaillard and Rouget have shown, that if the toxin is removed from the tetanus bacilli, they can then be introduced into an experimental animal without evil effects, because they are quickly taken up by phagocytes and destroyed; but when tetanus bacilli containing antitoxin are introduced they are not taken up by phagocytes, and quickly produce toxin, causing the development of tetanus. The antitoxin, injected immediately after the toxin-containing bacilli are introduced into the body, neutralizes their poison to such an extent that they can be destroyed by the phagocytes; but if the antitoxin is not present in the blood when the animal is inoculated with the germs of tetanus, or if it is not introduced into the circulation shortly thereafter, the tetanus bacilli produce so much toxin that it cannot be neutralized, and the development of tetanus cannot be prevented. That is to say, tetanus can be prevented with great certainty by the use of antitoxin; but when the symptoms of the disease have once appeared there is so much toxin in circulation that it cannot be neutralized by any amount of antitoxin that it is possible to introduce, so that the treatment of tetanus with antitoxin is not yet established on a profitable basis.

The prevention of the disease, however, by the use of antitoxin is in many instances practicable and a valuable resource. For example, it is known that tetanus is much more prevalent in some districts than in others, and that exposure is frequently followed by tetanus. It is also known that on some farms and in some stables tetanus is a common disease, and even slight injuries result in death from this cause. Under these conditions it may be serviceable to administer to the animal an immunizing dose of antitoxic serum, after which the contemplated operation may be performed without risk. Or if a valuable animal sustains a puncture of the foot or a punctured wound or a calk-injury, and particularly if the animal is kept in a stable where tetanus has previously appeared, it would be advisable as a general precautionary measure to protect it by the use of antitoxic serum.

My attention was recently called by Dr. Walters, of Wilkes-barre, to a mine in Dauphin County wherein there were almost one hundred mules, and of these twenty-three died of tetanus during a period of about two years. In January five or six died. I obtained some tetanus antitoxin, which was administered to fifty mules that occupied the stables in which the tetanus had previously appeared. Since that time no cases of tetanus have been reported.

Tetanus antitoxin has been used most extensively by Nocard in France, and Schutz in Germany, and they report that the mortality from tetanus has been considerably reduced by the use of this agent in districts where the disease prevails extensively and where antitoxin has been used before operations and after injuries as a precautionary measure.

The recent researches that have been made in relation to this disease, and the knowledge that we now have in reference to its toxin, explain the indifferent results that have attended the use of medicines. While it is true that certain therapeutic agents will, for a time, prevent the spasms that occur in tetanus, they cannot cure the disease, for they do not neutralize the poison, and as soon as the animal emerges from the effect of the drug the spasms are as bad as ever; but during the entire period the nervous system is irritated by the toxin it is gradually losing its vitality, and the animal is approaching death.

The only forms of treatment that are at all promising in the light of our present knowledge of this disease are: (1) The use of tetanus antitoxin; (2) the excision or sterilization of the local wound; (3) complete quietude and rest, thus giving an animal an opportunity to conserve its strength to the greatest possible degree, and make its conditions most favorable for overcoming the action of the toxin. It may be that other means of neutralizing the toxin will in time be discovered, or that it may become possible to produce the elimination of the toxin from the body; but as yet all efforts in this direction, including the use of purgatives, diuretics, and diaphoretics, have signally failed. Our greatest hope is in the development of the antitoxic treatment.

---

Chicago has a mounted brass band of thirty-five pieces.

